# HIV-1 Glycan Density Drives the Persistence of the Mannose Patch within an Infected Individual

**DOI:** 10.1128/JVI.01542-16

**Published:** 2016-11-28

**Authors:** Karen P. Coss, Snezana Vasiljevic, Laura K. Pritchard, Stefanie A. Krumm, Molly Glaze, Sharon Madzorera, Penny L. Moore, Max Crispin, Katie J. Doores

**Affiliations:** a Department of Infectious Diseases, Faculty of Life Sciences and Medicine, King's College London, London, United Kingdom; b Oxford Glycobiology Institute, Department of Biochemistry, University of Oxford, Oxford, United Kingdom; c Department of Virology, University of the Witwatersrand, Johannesburg, South Africa; d National Institute for Communicable Diseases (NICD) of the National Health Laboratory Service (NHLS), Johannesburg, South Africa; e Centre for the AIDS Programme of Research in South Africa (CAPRISA), University of KwaZulu Natal, Durban, South Africa; Ulm University Medical Center

## Abstract

The HIV envelope glycoprotein (Env) is extensively modified with host-derived N-linked glycans. The high density of glycosylation on the viral spike limits enzymatic processing, resulting in numerous underprocessed oligomannose-type glycans. This extensive glycosylation not only shields conserved regions of the protein from the immune system but also acts as a target for anti-HIV broadly neutralizing antibodies (bnAbs). In response to the host immune system, the HIV glycan shield is constantly evolving through mutations affecting both the positions and numbers of potential N-linked glycosylation sites (PNGSs). Here, using longitudinal Env sequences from a clade C-infected individual (CAP256), we measured the impact of the shifting glycan shield during HIV infection on the abundance of oligomannose-type glycans. By analyzing the intrinsic mannose patch from a panel of recombinant CAP256 gp120s displaying high protein sequence variability and changes in PNGS number and positioning, we show that the intrinsic mannose patch persists throughout the course of HIV infection and correlates with the number of PNGSs. This effect of the glycan density on the processing state was also supported by the analysis of a cross-clade panel of recombinant gp120 glycoproteins. Together, these observations underscore the importance of glycan clustering for the generation of carbohydrate epitopes for anti-HIV bnAbs. The persistence of the intrinsic mannose patch over the course of HIV infection further highlights this epitope as an important target for HIV vaccine strategies.

**IMPORTANCE** Development of an HIV vaccine is critical for control of the HIV pandemic, and elicitation of broadly neutralizing antibodies (bnAbs) is likely to be a key component of a successful vaccine response. The HIV envelope glycoprotein (Env) is covered in an array of host-derived N-linked glycans often referred to as the glycan shield. This glycan shield is a target for many of the recently isolated anti-HIV bnAbs and is therefore under constant pressure from the host immune system, leading to changes in both glycan site frequency and location. This study aimed to determine whether these genetic changes impacted the eventual processing of glycans on the HIV Env and the susceptibility of the virus to neutralization. We show that despite this variation in glycan site positioning and frequency over the course of HIV infection, the mannose patch is a conserved feature throughout, making it a stable target for HIV vaccine design.

## INTRODUCTION

The HIV envelope glycoprotein (Env) is coated in a dense array of host-derived N-linked glycans. These glycans not only shield conserved regions of the protein from neutralizing antibodies (nAbs), but also act as targets for many of the most broad and potent HIV neutralizing antibodies (1–6). Although HIV Env is glycosylated by the host cell glycosylation machinery, Env glycosylation has been shown to diverge from that typically observed in mammalian cells (1, 7–15). The dense clustering of potential N-linked glycosylation sites (PNGSs) sterically restricts the access of glycan-processing enzymes in the endoplasmic reticulum (ER), which results in a population of underprocessed oligomannose-type glycans (7–17) that is a distinctive feature of HIV Env (2) and is independent of producer cells (18). Site-specific analysis of the glycans on recombinant gp120 shows that these oligomannose-type glycans cluster together on the outer domain (OD) of gp120 (11, 13, 14, 19, 20), and this cluster is often referred to as the mannose patch and is conserved across Env expression systems (including virion-associated Env, SOSIP trimers, and recombinant gp120 monomers) and different geographical clades (7, 15, 17, 18, 20, 21). During expression of both monomeric and trimeric gp120, this mannose population is termed the “intrinsic mannose patch” (1, 2, 18). In the native trimer, in addition to the intrinsic mannose patch, further steric constraints on glycan processing give rise to the so-called “trimer-associated mannose patch” (1, 2, 18, 21, 22).

Three main glycan-dependent sites of vulnerability on Env have been identified so far. They include the N332 glycan/V3 loop, which comprises the intrinsic mannose patch (recognized by, e.g., PGT128, PGT121, 10-1074, and PGT135 [5, 23–25]), but also the N160 glycan/V1/V2 loops (recognized by, e.g., PG9, PG16, PGT145, CAP256-VRC26.25, and CH04 [5, 26–28]) and the glycans near the gp120/gp41 interface (recognized by, e.g., PGT151, 35O22, 8ANC195 [29–31]). Protein epitopes, such as the CD4 binding site and the membrane-proximal external region (MPER), also show some dependence on N-linked glycosylation. For example, the glycans situated on the rim of the CD4 binding site can modulate the neutralization breadth and potency of CD4 binding site broadly neutralizing antibodies (bnAbs) (22, 32), and the perturbation of gp41 glycosylation has been shown to influence the maximum neutralization of MPER bnAb 10E8 (33).

During infection, HIV Env is under constant pressure from the host immune system, in particular neutralizing antibodies, and as such, the location and frequency of PNGSs often change (3, 34). This observation has led to the concept of the shifting or evolving glycan shield (3). Recent studies aimed at mapping the development of HIV bnAbs in HIV-infected patients has revealed the importance of shifting PNGSs in bnAb development and has shown that immune escape from strain-specific antibodies can lead to formation of bnAb epitopes (35–37). For example, Moore et al. showed in an HIV-infected individual that immune pressure against the N334 glycan in a founder virus led to a shift to the conserved N332 glycan position and subsequent development of an N332-dependent bnAb response in that donor (35). Further, removal of the N276 glycan has been shown to confer sensitivity to germ line variants of CD4 binding site bnAbs, e.g., VRC01 and NIH45-46, indicating that addition of this glycan as a potential escape mechanism is critical for development of a broadly neutralizing CD4 binding site antibody response (38).

Studies comparing Env sequences from donor-recipient pairs and large numbers of acute and chronic viruses have shown that clade C transmitted viruses, and to a lesser extent clades A and D, tend to have shorter variable loops and a lower number of PNGSs than chronic viruses (39–43). These trends are observed for both sexual transmission and mother-to-child transmission; however, the significance of these differences for HIV transmission is not fully understood. Analysis of longitudinal Env sequences over years of HIV infection has shown that there is an increase in both variable-loop length and PNGS frequency, which is reversed in the later stages of infection (44, 45). It is proposed that this initial increased glycosylation shields neutralizing protein epitopes from the host immune system, which also wanes during late infection (3, 34, 46, 47). Although these studies defined changes in the position and frequency of PNGSs over the course of HIV infection, the effects of these changes on the composition of the glycans present on Env, in particular the persistence of the intrinsic mannose patch, have not yet been determined.

Here, we use longitudinal Env sequences from a clade C-infected donor, CAP256, to determine the change in glycan shield composition and the abundance of oligomannose-type glycans in the intrinsic mannose patch over the course of HIV infection and to relate these changes to variable-loop length, frequency of PNGSs, and neutralization sensitivity by a panel of HIV bnAbs. The development of the bnAb response in donor CAP256 has been extensively studied and is mediated by bnAbs directed to the V1/V2 region on Env (27, 48, 49). This patient was infected with a clade C virus and later became superinfected with a second, unrelated clade C virus between weeks 13 and 15, leading to Env recombination (27, 48, 49). The viral population early in infection was predominantly made up of the superinfecting (SU) virus with only the V1/V2 and gp41 C terminus mostly derived from the primary infecting (PI) virus (49), but later in infection, multiple different recombinant forms existed. Escape from the bnAb response occurred through mutation in V2, in particular at residues R166 and K169 (27, 48, 49).

Here, we show that although the number of PNGSs varies by up to five, the intrinsic mannose patch is conserved across all gp120 proteins. However, we observed variations in both the size and composition of the intrinsic mannose patch. We show that there is a strong correlation between the frequency of outer-domain PNGSs and the abundance of oligomannose-type glycans for both CAP256 gp120s and a cross-clade panel of gp120s, highlighting the importance of the glycan density for the restricted access by glycan-processing enzymes. Although there were no strong correlations across the full time period in this donor, a general increase in total PNGSs was observed early in infection, and this increase correlated with an increase in oligomannose-type glycans. This was followed by a decline in PNGSs due to loss of glycans at the V3 base and a subsequent decline in oligomannose-type glycans, which was associated with the development of neutralizing antibodies to the C3V4 region. These results demonstrate the persistence of the intrinsic mannose patch over the course of HIV infection and further highlight this region as a stable target for HIV vaccine design strategies.

## MATERIALS AND METHODS

### Cloning and protein production.

Cloning of the full-length soluble ectodomain of HIV-1 CAP256 gp120s (corresponding to amino acid residues 1 to 507, based on alignment to the HxB2 reference strain) into the pHLsec expression vector (50) has been described previously (16, 51). The CAP256 Env sequences were published previously (48, 49). The CAP256 proteins were expressed in the 293F variant of HEK 293T cells (ThermoFisher Scientific), which is adapted for suspension culture, in 500-ml Erlenmeyer flasks with a vent cap (Corning). The cells were incubated at 37°C and 5% CO_2_ with shaking at 137 rpm as recommended by the manufacturer. Briefly, 200-ml cultures were transfected with plasmids (pHLSec) carrying the reporter gene expressing the protein using 293Fectin (ThermoFisher Scientific). The culture supernatants were harvested 5 days after transfection, and the His-tagged proteins were purified by Ni^2+^ affinity purification using a 5-ml HisTrap FF column (GE Healthcare). The nickel-purified proteins were further purified using size exclusion chromatography (SEC) on a Superdex 200 16/600 column (GE Healthcare). The monomeric fractions were collected, pooled, and analyzed using an SDS-PAGE 4% to 12% Bis-Tris NuPAGE gel (Invitrogen).

### Glycan profiling by PNGase F release of N-glycans.

N-glycans were released from target glycoprotein immobilized in SDS-PAGE bands using peptide-*N*-glycosidase F (PNGase F) (New England BioLabs) (52). Coomassie-stained gel bands were excised and washed alternately with acetonitrile and water before being dried under vacuum. The gel pieces were rehydrated in 20 mM sodium bicarbonate buffer, pH 7.0, and incubated with PNGase F (1 μl) for 16 h at 37°C. The released glycans were extracted from the gel matrix by 3 washing steps with water.

### Fluorescent labeling of N-linked glycans.

The released glycans were subsequently fluorescently labeled and purified as previously described (53). The PNGase F-released N glycans were fluorescently labeled using 2-aminobenzoic acid (2-AA). The labeling mixture comprised 2-AA (30 mg/ml) and sodium cyanoborohydride (45 mg/ml) dissolved in a solution of sodium acetate trihydrate (4% [wt/vol]) and boric acid (2% [wt/vol]) in methanol. The labeling mixture (80 μl) was added to each sample (in 30 μl of water) and incubated at 80°C for 1 h. The labeled oligosaccharides were purified using Spe-ed amide-2 columns (Applied Separations, Allentown, PA) preequilibrated with acetonitrile. Before loading, 1 ml 97% (vol/vol) acetonitrile was added to each sample. The loaded samples were then washed with 2 ml 95% (vol/vol) acetonitrile and eluted with 1.5 ml water. The glycans were dried under vacuum prior to ultraperformance liquid chromatography (UPLC) analysis or glycosidase treatment.

### Digestion of free labeled glycans.

Glycan samples labeled with 2-AA were digested overnight using endoglycosidase H (Endo H) (New England Bioscience) in a total volume of 20 μl. Samples were purified with a protein-binding membrane cleanup, using a Ludger vacuum manifold and a multiscreen filter protein-binding plate (Millipore).

### Hydrophilic interaction liquid chromatography-ultraperformance liquid chromatography.

Glycans were separated by hydrophilic interaction liquid chromatography (HILIC)-UPLC using a Waters Acquity system (Waters, USA). The labeled samples were resuspended in 15 μl water and added to a vial with 15 μl 100% acetonitrile. A 2.1-mm by 10-mm Acquity BEH amide column (Waters; particle size, 1.7 μm) with a programmed gradient was used for separation. Data were acquired and processed with Empower 3 (Waters, USA).

### Pseudovirus production and neutralization assays.

To produce pseudoviruses, plasmids encoding Env were cotransfected with an Env-deficient genomic backbone plasmid (pSG3ΔEnv) in a 1:2 ratio with the transfection reagent polyethyleneimine (PEI) (1 mg/ml; 1:3 PEI-total DNA; Polysciences) into HEK 293T cells (obtained from the American Type Culture Collection) (54, 55). Pseudoviruses were harvested 72 h posttransfection for use in neutralization assays. Neutralizing activity was assessed using a single-round replication pseudovirus assay with TZM-bl target cells (provided by John Kappes through the NIH AIDS Reagents Repository Program), as described previously (54, 55). Briefly, the antibody was serially diluted in a 96-well flat-bottom plate and preincubated with virus for 1 h at 37°C. Cells at a concentration of 20,000/well were added to the virus-antibody mixture, and the luminescence was quantified 72 h following infection via lysis and addition of Bright-Glo Luciferase substrate (Promega). Dose-response curves were fitted using nonlinear regression (GraphPad Prism) to determine 50% inhibitory concentrations (IC_50_s).

### Antibodies.

PGT121, PGT128, PGT135, PG9, PGV04, VRC01, PGT151, and CAP256-VRC26.25 were transiently expressed with the FreeStyle 293 expression system (Thermofisher Scientific). The antibodies were purified using affinity chromatography (Protein A Sepharose Fast Flow; GE Healthcare), and the purity and integrity were checked by SDS-PAGE.

### Correlations and statistics.

Correlations were determined using a Pearson correlation and calculated using GraphPad Prism 6.

### Preparation of chimeric viruses.

Chimeric Env containing the C3V4 region were created using an overlapping PCR strategy and cloned into the pCDNA 3.1D-TOPO vector (Invitrogen) as described previously (56). The chimeric viruses were used to generate pseudoviruses as described above and assayed for neutralization sensitivity to longitudinal CAP256 plasma (obtained from the CAPRISA cohort). Site-directed mutagenesis was used to delete the N332 glycan within this construct to assess the role of the glycan in mediating escape from plasma nAbs.

## RESULTS

### Longitudinal analysis of PNGSs and V loop lengths for CAP256 sequences.

**FIG 1:**
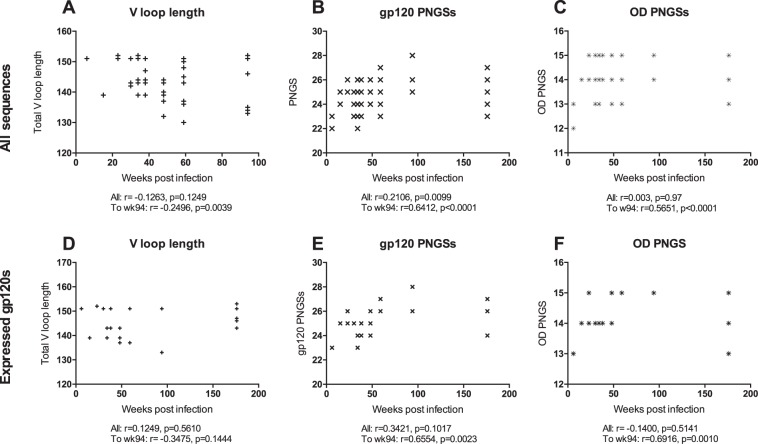
(A to C) Correlation between number of weeks postinfection and total length of variable loops (number of amino acids) (V1 to V5) (A), total number of PNGSs on gp120 (B), and number of PNGSs on the gp120 outer domain (residues 252 to 482) (C). A total of 154 previously published Env sequences over multiple time points were used in the analysis (48). (D to F) Correlation between the number of weeks postinfection and the total length of variable loops (V1 to V5) (D), the total number of PNGSs on gp120 (E), and the number of PNGSs on the gp120 outer domain (residues 252 to 482) (F) for the 24 recombinantly expressed gp120s. The PI virus and SU virus were at weeks 6 and 15, respectively. Correlations were assessed by Pearson analyses; *P* values and *r* values are indicated between weeks 6 and 94 and between weeks 6 and 176 (All). Note that some of the sequences have identical PNGS, OD PNGS, and V loop lengths, and these points are overlaid.

**FIG 2:**
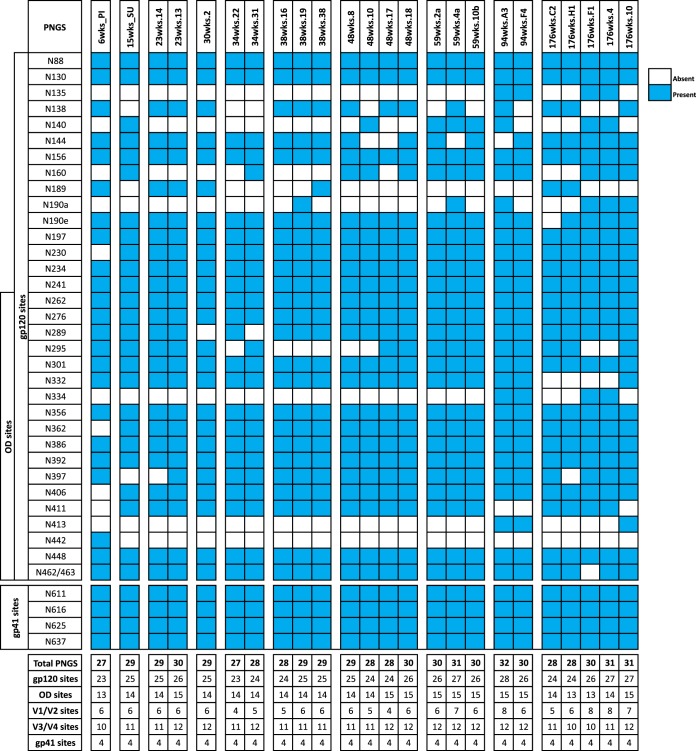
Summary of PNGSs for 24 representative CAP256 Env clones that were expressed recombinantly. The total number of PNGSs on gp160, gp120, the outer domain, V1/V2, V3/V4, and gp41 were calculated for each clone. Clones are grouped together according to the time they were isolated.

Env sequences from the CAP256 donor from multiple time points over the course of HIV infection have been reported previously (48, 49). Full Env single-genome amplification (SGA) and next-generation sequencing of the V1-to-V3 region (using the MiSeq platform) of viral variants from plasma samples correlated well (48, 57). Here, 154 clones from multiple time points were analyzed for their PNGS positions and frequencies, as well as their variable-loop lengths. Previous studies have reported an increase in PNGSs and variable-loop lengths over the course of HIV infection (44, 45). Therefore, we first determined whether these trends were observed in CAP256 (Fig. 1). We first considered the changes in variable-loop lengths over time. Although there was variation in both the individual and total variable-loop lengths during the course of infection, there were no notable correlations. However, there was a weak negative correlation between total V loop length and the number of weeks postinfection until week 94 (*r* = −0.2496; *P* = 0.0039) (Fig. 1A). We next considered PNGS frequency. For all CAP256 Env sequences, the frequency of gp41 PNGSs remained constant at 4, and the locations of these sites did not change during the course of infection (Fig. 2). The frequency of total PNGSs for gp120 ranged from 22 to 28, with the PI and SU viruses having mostly 23 and 25 PNGSs, respectively (Fig. 1B). The majority of variation in PNGS frequency occurred within variable loops, in particular the V1/V2 loops and glycan sites positioned at the base of the V3 loop (N295, N332, and N334). When the frequency of PNGSs was plotted against the number of weeks postinfection, a weak positive correlation (*r* = 0.21; *P* = 0.01) was observed. However, as the glycan shield is a dynamic entity that is under constant pressure from the host immune system, we also looked for correlations over shorter periods. In this donor, a strong positive correlation (*r* = 0.64; *P* < 0.0001) was observed until week 94, after which the number of PNGSs declined and the correlation weakened (Fig. 1B). This decrease corresponded predominantly to the loss of glycan sites at positions N295 and N332. A slight decrease in PNGSs around weeks 30 to 34 was also observed, which corresponded to loss of V1/V2 loop PNGSs and the N289 or N295 glycan sites (Fig. 1B). Interestingly, this is the first time point at which the V1/V2-specific antibody response was detected and subsequently led to a sudden increase in viral diversification (48, 57). A similar trend was observed for PNGSs on the OD (residues 252 to 482) of gp120 until week 94; however, there was no correlation over the full period (Fig. 1C). In summary, in the CAP256 donor, there was a general trend toward increasing numbers of PNGSs early in infection that decreased at the latest time point (week 176), which is consistent with previous studies (44, 45). However, there was still considerable variation between single viruses at a given time point (e.g., at week 176, the total numbers of PNGSs differed by 4 [Fig. 1B]), enabling us to assess the prevalence of the intrinsic mannose patch.

### The intrinsic mannose patch is present on gp120 throughout HIV infection in donor CAP256.

**FIG 3:**
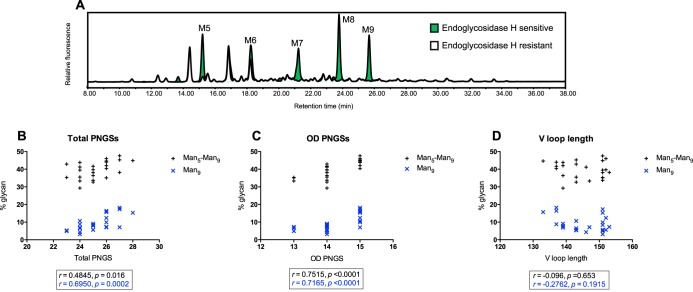
(A) HILIC-UPLC spectrum of fluorescently labeled N-linked glycans released from 48wks.17 gp120 using PNGase F presented as an example of the quantification methodology. The green trace is a spectrum of released glycans, and the white trace is the spectrum for Endo H-treated glycans. Overlaying of the spectra resulted in the glycans sensitive to Endo H being displayed as green. M5–M9 refers to Man_5–9_GlcNAc_2_. (B to D) The percentage of oligomannose glycans was assessed by integration of chromatograms pre- and post-Endo H digestion, generating specific percentage areas for the oligomannose glycans. The oligomannose glycans are highlighted. Shown are correlations between the abundances of oligomannose-type glycans (Man_5–9_GlcNAc_2_ and Man_9_GlcNAc_2_) and total PNGSs on gp120 (B), PNGSs on the gp120 outer domain (residues 252 to 482), and total variable-loop lengths (D). The correlations were assessed by Pearson analyses; *P* values and *r* values are indicated.

To determine changes in the composition of the HIV glycan shield and the abundance of oligomannose-type glycans within the intrinsic mannose patch over the course of infection, the gp120 regions of 24 CAP256 Envs from different time points were recombinantly expressed. The selected clones were chosen to represent major clades within a phylogenetic tree based on single-genome amplification. This smaller sample of CAP256 Envs displayed a correlation between the number of weeks postinfection and PNGS frequency similar to that for the 154 Env sequences (Fig. 1D to F), and their PNGS positions and frequencies are shown in Fig. 2. We were particularly interested in the abundance of oligomannose-type glycans of the intrinsic mannose patch, as these glycans form part of the epitopes of a number of the most broad and potent HIV bnAbs (e.g., PGT121, PGT128, and PG9) (5, 25, 58, 59). As we have previously shown that the intrinsic mannose patch of recombinant gp120 captures much of the steric constraints exhibited by these glycans in the context of the trimer (including SOSIP trimers) (7, 15, 17, 18, 22), monomeric gp120 was used as a useful model of this viral feature. Residues 1 to 517 were cloned into a recombinant expression vector (pHLSec) (50, 51) and expressed in HEK 293F cells for glycan profiling (we have previously shown that the mannose patch is largely independent of the producer cell [17, 18]). The protein constructs included a C-terminal hexahistidine tag so that nickel affinity purification could be used to avoid potential bias associated with other glycan-specific purification methods, such as lectins. Proteins were purified first using His tag affinity chromatography, followed by SEC to remove aggregates. The purified proteins were then run on a nonreducing SDS-PAGE gel, and the monomeric gp120 band was excised for glycan analysis. N-linked glycans were released using PNGase F, fluorescently labeled, and analyzed by HILIC-UPLC. The percentage of oligomannose glycans was assessed by integration of chromatograms pre- and post-Endo H digestion, generating specific percentage areas for the oligomannose glycans (Fig. 3A). It was then possible to assign structures based on previous analysis (16, 18).

All gp120 samples displayed an intrinsic mannose patch; however, the population of oligomannose-type glycans varied from 29.3% to 47.6% (see Table S1 in the supplemental material). The percent changes in oligomannose levels between gp120 94wks.A3, which has the highest number of PNGSs (a total of 28), and 6wks_PI, which has the fewest PNGSs (a total of 23), are 27% and 212% for Man_5–9_GlcNAc_2_ and Man_9_GlcNAc_2_, respectively. gp120 94wks.A3 has additional PNGSs in V1 (N135 and N160), in C2 (N230), in C3 (N362), and in V4 (N406, N413), whereas 6wks_PI has an additional PNGS in C4 (N442). We have previously measured the decrease in oligomannose-type glycans on BaL gp120 when one or two PNGSs were removed through Asn-to-Ala substitution (16). The largest effect was observed for the N295A/N386A double mutant, where the percentages of Man_5–9_GlcNAc_2_ and Man_9_GlcNAc_2_ decreased by 27% and 71%, respectively. Therefore, compared to our previous observations, the differences in oligomannose-type glycan abundances for 94wks.A3 and 6wks_PI are relatively small considering that these recombinant proteins differ by five PNGSs (16), but the difference in Man_9_GlcNAc_2_ structures is much higher. This observation was not unexpected, given that the positions of two of the additional PNGSs are on the gp120 OD, where PNGSs are tightly clustered (see Discussion below). This therefore suggests that there are regions on gp120 where multiple glycans can be removed with little impact on glycan processing of the intrinsic mannose patch and that it is the local density of PNGSs that determines the extent of glycan processing.

### The abundance of oligomannose-type glycans correlates with the density of PNGSs.

To determine factors that might influence the abundance of specific oligomannose-type glycans on gp120, we correlated the percentage of Man_5–9_GlcNAc_2_ glycans with the total number of PNGSs on gp120 (Fig. 3B). A positive correlation was observed (*r* = 0.486; *P* = 0.016), and this correlation became more significant when only Man_9_GlcNAc_2_ glycan abundance was considered (*r* = 0.695; *P* = 0.0002). When the percentages of Man_5–9_GlcNAc_2_ and Man_9_GlcNAc_2_ were correlated with the frequency of PNGSs present only on the OD of gp120, a strong positive correlation was observed for both Man_5–9_GlcNAc_2_ and Man_9_GlcNAc_2_ (*r* = 0.752, *P* = < 0.0001, and *r* = 0.717, *P* = < 0.0001, respectively) (Fig. 3C). The gp120 OD PNGSs include many of the sites shown to be oligomannose type in site-specific analysis studies (8, 11, 14, 22, 60). Further, the recent crystal structures of the BG505 SOSIP.664 recombinant trimer showed that the PNGSs in the region cluster tightly on the surface of Env (58, 61). Therefore, an increase in PNGSs on the outer domain of gp120 will likely further restrict access of the glycan-processing enzymes, leading to an increase in oligomannose-type glycans on gp120 and in the size of the intrinsic mannose patch (7, 17). Interestingly, some gp120s that have the same positioning and frequency of PNGSs still showed differences in the percentages of oligomannose-type glycans, highlighting the role the protein sequence may also play in determining the structure of the HIV glycan shield. For example, 59wks.2a and 59wks.10b have identical PNGSs, yet the percentages of oligomannose-type glycans differ by 3.6%.

Longer variable-loop lengths might be expected to decrease the density of PNGSs and lead to a higher degree of glycan processing and therefore to a reduction in oligomannose-type glycans. Although the CAP256 gp120 sequences differed in combined variable-loop lengths by up to 20 amino acids, no correlation was observed between oligomannose abundance and variable-loop length (Fig. 3D), suggesting that the positioning of specific glycan sites is most critical to oligomannose abundance.

### The abundance of oligomannose-type glycans correlates with the density of outer-domain PNGSs for a cross-clade panel of gp120s.

**FIG 4:**
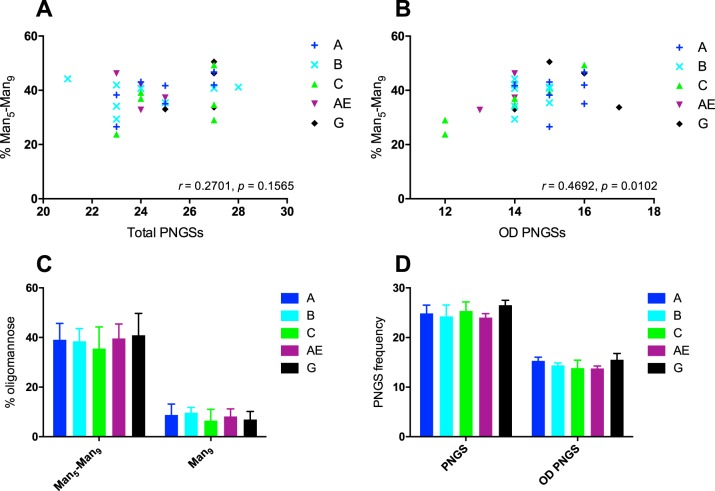
(A and B) Correlation between the percentage of oligomannose-type glycans (Man_5–9_GlcNAc_2_) and total number of PNGSs on gp120 (A) and PNGSs on the outer domain of gp120 (residues 252 to 482) (B) for a cross-clade panel of gp120 glycoproteins. Each point is colored depending on the HIV clade (A, *n* = 7; B, *n* = 8; C, *n* = 6; AE, *n* = 4; and G, *n* = 4). The correlations were assessed by Pearson analyses; *P* values and *r* values are indicated. (C and D) Cross-clade gp120 panel differences for Man_5_-Man_9_ and Man_9_ (C) and total PNGSs and OD PNGSs (D). The error bars represent standard deviations. A Mann-Whitney test was used to show that there were no significant differences between the groups.

To determine whether the correlation between the number of PNGSs on the outer domain of gp120 and the abundance of oligomannose-type glycans was a general feature for HIV Env across geographical clades, a panel of 29 gp120s were cloned, expressed, and purified as described above. The panel included gp120s from clades A, B, C, AE, and G, five of which were transmitted/founder viruses. All the isolates tested were found to possess a significant population of oligomannose-type glycans ranging from 23.8% to 50.5% (Fig. 4; see Table S2 in the supplemental material). When the abundance of oligomannose-type glycans was correlated with the total number of PNGSs (ranging from 21 to 28), no significant correlation was observed (Fig. 4A). However, a significant correlation was observed between the frequency of OD PNGSs (ranging from 12 to 17) and oligomannose abundance, similar to that seen for the CAP256 samples (*r* = 0.4692; *P* = 0.010) (Fig. 4B). No significant correlations were observed between total PNGSs or OD PNGSs and Man_9_GlcNAc_2_ (data not shown). These data further support the notion that a high density of PNGSs on the OD restricts glycan-processing enzymes, leading to a larger population of underprocessed oligomannose-type glycans. These data also suggest that it is local glycan density that has the largest impact on glycan processing, rather than overall glycan density. Interestingly, the specific occupancy and composition of individual sites was not assessed here, but this could be an informative extension in future studies.

While there are variations in the percentages of certain oligomannose structures between the clades (clades C and G have fewer Man_9_GlcNAc_2_ structures, and clade C has fewer Man_8_GlcNAc_2_ structures), the overall abundances of oligomannose glycans are fairly similar (Fig. 4C and D). Clade C has the lowest total percentage of oligomannose (35.5%), yet compared to clade B, with one of the highest percentages and lowest standard deviation (SD) (38.5%; SD, 4.98), there is no significant difference between the two (Fig. 4C), although this difference might become more significant if more gp120s were studied. Considering the correlation between outer-domain PNGSs and Man_5–9_GlcNAc_2_, it is likely that loss of specific sites between clades is responsible for the differences in specific glycan abundance. This is particularly relevant for clade C viruses, which typically lack the N295 glycan site (62, 63), a PNGS we have previously shown to stabilize the mannose patch from glycan processing (16). However, while there are some differences in the structures, the total levels of oligomannose-type glycans remain similar between clades, indicating the overall stability and conserved nature of the mannose patch.

### Correlation of oligomannose-type glycans with time postinfection.

**FIG 5:**
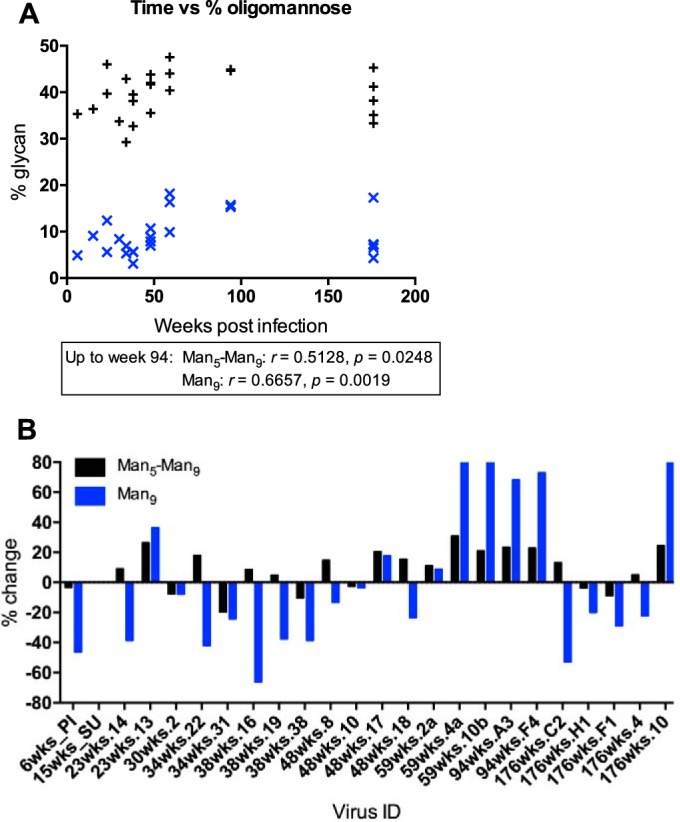
(A) Correlation between number of weeks postinfection and abundances of Man_5–9_GlcNAc_2_ and Man_9_GlcNAc_2_. (B) Percent change in Man_5–9_GlcNAc_2_ and Man_9_GlcNAc_2_ compared to the SU virus for each CAP256 gp120 clone studied {percent change = [(percent CAP − percent SU)/percent SU] × 100}. The correlations were assessed by Pearson analyses; *P* values and *r* values are indicated.

We next examined how the size of the intrinsic mannose patch changes over the course of HIV infection. We first correlated the percentage of oligomannose-type glycans with the number of weeks postinfection, but no correlation was observed (Fig. 5A). As the glycan shield is a dynamic entity that is under constant pressure from the host immune system, we also looked for correlations over shorter periods to reflect this. We observed correlations between Man_5–9_GlcNAc_2_ and Man_9_GlcNAc_2_ abundance and the number of weeks postinfection until week 94 (*r* = 0.513, *P* = 0.025, and *r* = 0.666, *P* = 0.0019, respectively) (Fig. 5A) similar to that seen for changes in PNGS frequency over time. The abundance of oligomannose-type glycans, then, largely persists but exhibits some variation due to sensitivity to loss of PNGSs at the base of V3, in particular at positions N295 and N332/N334 (Fig. 2).

To analyze the changes in Env glycan composition over time in more detail, we next determined the percent change in total oligomannose-type glycans (Man_5–9_GlcNAc_2_) and Man_9_GlcNAc_2_ individually for each gp120 clone (Fig. 5B). As the viral population early in infection was predominantly made up of the SU virus, with only the V1/V2 and gp41 C terminus mostly being derived from the PI virus, changes in total Man_5–9_GlcNAc_2_ and Man_9_GlcNAc_2_ composition were considered in relation to the SU virus. Although the mannose patch is present on all CAP256 proteins studied, the changes in Man_5–9_GlcNAc_2_ and Man_9_GlcNAc_2_ can vary for individual clones at a given time point. Generally, a large increase in oligomannose-type glycans was due to increases in Man_8_GlcNAc_2_ and Man_9_GlcNAc_2_ early-stage glycan structures (Fig. 5B; see Table S1 in the supplemental material), further suggesting that increased density in PNGSs leads to reduced glycan processing. For example, gp120s from weeks 59 and 94, which had the highest oligomannose-type glycan abundance (44.0 to 47.6%), had 28.4% to 30.4% Man_8–9_GlcNAc_2_ structures. Interestingly, previous analysis of glycan site mutants showed that the presence of Man_9_GlcNAc_2_ was particularly dependent on multiple stabilizing interactions with neighboring glycans (16).

### The abundance of oligomannose-type glycans does not correlate with the neutralization potency of anti-HIV bnAbs.

**FIG 6:**
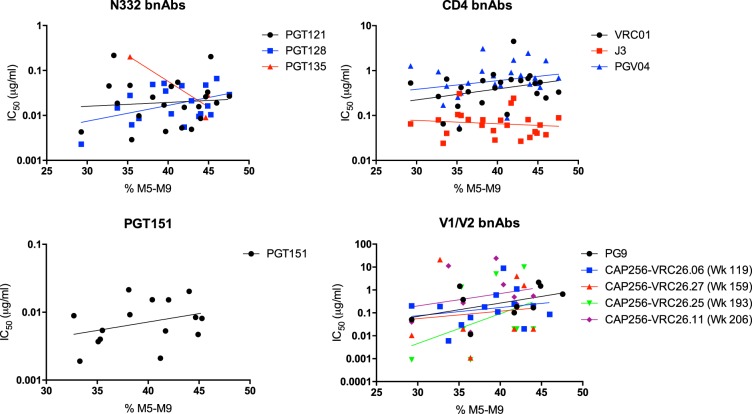
Correlation between the potency of neutralization (IC_50_s) and the percentage of oligomannose-type glycans (M5–M9, Man_5–9_GlcNAc_2_) for a panel of HIV bnAbs: N332-dependent (intrinsic mannose patch binding) bnAbs, CD4 binding site bnAbs, PGT151, and N160 V1/V2 loop bnAbs. The correlations were assessed by Pearson analyses. The IC_50_s are listed in Table S3 in the supplemental material.

We next wanted to determine whether the structure of the HIV glycan shield, in particular the abundance of oligomannose-type glycans, might influence the potency of neutralization by a panel of HIV bnAbs. We therefore determined the IC_50_s for the intrinsic mannose patch binding bnAbs PGT121, PGT128, and PGT135; the V1/V2 loop bnAb PG9 and several members of the CAP256-VRC26 antibody lineage; cleavage-specific bnAb PGT151; and CD4 binding site bnAbs PGV04, VRC01, and llama antibody VHH J3 (see Table S3 in the supplemental material). When the IC_50_s were correlated with the abundances of oligomannose-type glycans, no significant correlations were observed for any bnAbs (Fig. 6), although a general weak trend for increasing IC_50_s with increasing oligomannose-type glycans was observed for some bnAbs. Generally, the ability of a bnAb to neutralize a viral variant was dependent on the presence of key contact glycan sites, such as N160 or N332. The majority of viruses were resistant to PGT135 neutralization, and viruses lacking the N332 glycan site, and in one case the N295 glycan site, were resistant to PGT128. PGT121 was able to neutralize all but two viruses. PG9 could not neutralize viruses lacking the N160 glycan site or viruses with a glutamic acid at position 169 (Fig. 2 and 6; see Table S3 in the supplemental material) (48), whereas CAP256-VRC26 lineage bnAbs were dependent on protein residues in V1 for neutralization (27). Interestingly, none of CAP256-VRC26 lineage bnAbs isolated over several different time points throughout infection (weeks 119, 159, 193, and 206) showed any correlation with oligomannose abundance, suggesting that increasing the size of the mannose patch is not a direct mechanism of escape against the autologous antibodies in this donor. For PGT151, although all the viruses contained the key glycan sites and residues thought to be required for neutralization (N611, N637, and E647), some viruses were nonetheless resistant to PGT151 neutralization. The potencies for the CD4 binding site bnAbs PGV04, VRC01, and J3, which do not contact glycans, generally did not correlate with the levels of oligomannose-type glycans. As N-linked glycans are positioned around the edge of the CD4 binding site, the changes in bulk glycan structures observed may not occur in this region of gp120 and therefore may not impact CD4 binding site bnAbs, but site-specific glycan analysis would be required to determine this. Interestingly, the smaller single-chain llama antibody, J3, had the smallest variation in IC_50_s. Therefore, the abundance of oligomannose-type glycans in the intrinsic mannose patch does not impact the potency of neutralization and suggests Env sequences from any time point during infection, provided they have the key contact glycan sites, would be suitable HIV immunogens.

### Anti-C3V4 nAbs may be responsible for loss of PNGSs at week 176.

**FIG 7:**
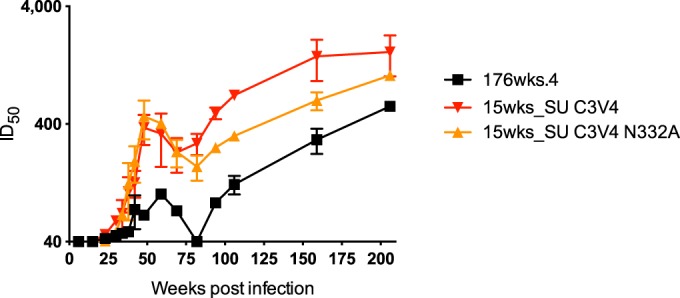
Kinetics of the C3V4 neutralizing antibody response in CAP256. Titers are shown using the CAP256 176wks.4 virus and chimeric Envs containing only the C3V4 region of the sensitive SU virus (15wks_SU C3V4) and an N332A variant (15wks_SU C3V4 N332A). Anti-C3V4 antibodies at titers greater than 1:100 were detected from 42 weeks postinfection and persisted at least until 94 weeks postinfection. The anti-C3V4 antibodies show some N332A dependence. Titers are indicated as plasma 50% infective dose (ID_50_) versus number of weeks postinfection.

The loss of glycan sites at the base of the V3 loop at week 176 suggested that neutralizing antibodies might exert selection pressure against this region that leads to loss of PNGSs and a decrease in abundance of oligomannose-type glycans. To assess whether the region was a target of nAbs, we created a chimeric Env from the 176wks.4 Env, which had already escaped the high-titer V2 responses that dominate CAP256 plasma (49). Using overlapping PCR, we transferred the C3V4 region from the sensitive 15wks_SU virus into the resistant backbone and tested this chimeric Env (15wks_SU C3V4) against longitudinal plasma (Fig. 7). Anti-C3V4 antibodies at titers greater than 1:100 were detected from 42 weeks postinfection, persisting at least until 94 weeks, at which time point an additional specificity emerged. To determine whether the anti-C3V4 antibodies were directed against the N332 glycan site in particular, we next used site-directed mutagenesis to make an Asn-to-Ala substitution at the N332 glycan site (15wks_SU C3V4 N332A). A decrease in serum titers was observed, indicating that some of the C3V4 antibody response is directed against the N332 epitope (Fig. 7). The presence of anti-C3V4 nAbs suggests that nAbs against the region can elicit a selective pressure that results in loss of V3 loop glycans and a subsequent decrease in oligomannose-type glycans.

## DISCUSSION

**FIG 8:**
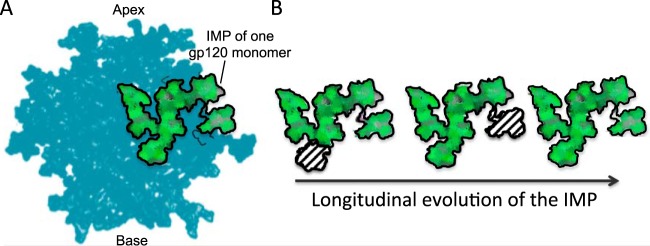
Schematic representation of the evolving HIV glycan shield. (A) Structure of BG505 SOSIP.644 trimer showing the presence of the intrinsic mannose patch (IMP) (green) present on one of the three gp120 monomers (22, 72). (B) Cartoon representation of the longitudinal evolution of the intrinsic mannose patch on gp120. Despite the changes in position and frequency of PNGSs on gp120, the intrinsic mannose patch persisted throughout infection in this individual. The green area represents the intrinsic mannose patch from one gp120 monomer.

It is clear that the HIV glycan shield is under constant pressure from the host immune system. Here, we used longitudinal Env sequences from a chronically infected HIV patient to characterize the changes in the structure of the HIV glycan shield during the course of HIV infection, in particular the persistence and composition of the intrinsic mannose patch. We showed that in the CAP256 donor, the mannose patch (Fig. 8A) persists throughout infection despite the variation in PNGS position and frequency (Fig. 8B). In this donor, there is an increase in PNGSs and oligomannose-type glycans within the intrinsic mannose patch over the course of infection until week 94. This increase correlates with the frequency of PNGSs on the outer domain. Thereafter, there is a reduction in PNGSs at the base of V3 and a corresponding reduction in oligomannose-type glycans by week 176, likely a consequence of viral escape from a *de novo* neutralizing response to the C3V4 region. Although this study focuses on only one donor, these findings give insight into the composition and conservation of the intrinsic mannose patch under immune pressure and highlight the epitope as an important target for HIV vaccine design strategies.

Our previous studies have shown that the glycosylation of HIV Env is determined by both protein-directed effects, arising from the 3-dimensional protein structure, and cell-directed effects, arising from the cell type the protein is expressed in (2, 17, 18). The protein-directed effects give rise to a patch of underprocessed oligomannose-type glycans on the outer domain of gp120 that forms a nonself epitope targeted by HIV bnAbs. We show that despite the variation in the protein sequence and the positioning and frequency of PNGSs, the intrinsic mannose patch is highly conserved during the course of infection in the CAP256 donor and therefore represents a stable target for vaccine design. However, the intrinsic mannose patch varies in both overall size and distribution of glycans within the oligomannose series (Man_5–9_GlcNAc_2_), and this most strongly correlates with the density of PNGSs present on the OD of gp120. This trend was also observed, although to a lesser extent, for a cross-clade panel of gp120s and highlights the role the protein sequence might also play in determining the structure of the HIV glycan shield. These data support our previous conclusions that the high density of PNGSs restricts glycan-processing enzymes from trimming and processing N-linked glycans within the region (7, 15, 16, 18, 64). Interestingly, it seems to be the local glycan density rather than the overall glycan density that has the biggest impact on the size and composition of the mannose patch. Although Env sequences vary by up to 5 PNGSs, it is clear that it is mainly PNGSs within and around the outer domain of gp120 that affect the size and distribution of oligomannose-type glycans within the intrinsic mannose patch. The potency of neutralization by a panel of HIV bnAbs is not affected by the variation in mannose patch composition but is dependent on the presence of certain key PNGSs. This suggests that PNGSs on gp120 have sufficiently high density that the natural variation in Env occurring throughout infection has minimal impact on glycan processing, so that the mannose patch, which is intrinsic to both the monomer and the trimer, is always present. Therefore, the density of glycans on gp120, even at the lowest density, is sufficient to maintain the steric restriction necessary to impede mannosidase processing. This is consistent with previous observations suggesting that minimal glycan-glycan interactions are required to prevent processing to complex-type glycans (16). In addition, this effect may be further compounded by the trimer-associated restriction of processing not captured by our monomeric gp120 model (1, 2, 18, 22). Although several studies have reported more compact transmitter clade C and A viruses (39, 40), with shorter V1-to-V4 loop lengths, this does not appear to impact the glycosylation of gp120s from the CAP256 donor.

The most dramatic changes to the HIV glycan shield of CAP256 gp120 occur when glycans at the base of the V3 loop are added or deleted. This is supported by our previous studies showing that deletion of glycans within this region for gp120_BaL_ had the largest impact on oligomannose-type glycan abundance due to disruption of glycan microclusters within the outer domain (16). We have shown that some of the changes occurring in the PNGS position and frequency of CAP256 gp120, and subsequently oligomannose abundance, at week 176 postinfection are likely a result of a new wave of neutralizing antibodies targeting the C3V4 region, including the N332 glycan. These data may suggest that the selective pressure of neutralizing Abs targeting the intrinsic mannose patch would have the biggest effect on shaping the glycan structures present on the HIV glycan shield. Unfortunately, full-length Envs from later time points were not available, but as the C3V4-specific response arose after approximately 75 weeks, any additional destabilization of the intrinsic mannose patch is likely to occur within the time frame studied. It is possible that if a similar study were carried out in a donor who developed bnAbs against another epitope, such as the CD4 binding site, less variation in OD PNGS frequency would occur, and thus, a smaller variation in oligomannose-type glycans would be observed over the course of infection.

Go and colleagues have previously compared the glycosylation of recombinant gp120 from transmitted/founder (t/f) viruses and chronic viruses (60). They concluded that t/f virus Envs are more similar to each other than to those of their corresponding chronic viruses, with t/f Envs having distinct glycosylation patterns consisting of higher levels of oligomannose and sialylated glycans and a lower site occupancy (60). However, the study was limited, as only two t/f and two chronic viruses were studied, and these viruses were not derived from the same donors. Indeed, comparison of oligomannose levels on the t/f and chronic viruses in our gp120 panel showed no significant differences. By using longitudinal virus sequences, we were able to show that over the course of infection in the CAP256 individual, there was an increase in PNGSs and a corresponding increase in oligomannose-type glycans that were subsequently reduced by the pressure of neutralizing antibodies. Although the PI and SU virus gp120s have lower levels of oligomannose-type glycans (35.3% and 36.4%, respectively) than the majority of gp120s from later time points, there are viruses within the quasispecies that have lower levels of oligomannose glycans, e.g., 38wks.38 and 48wks.10, with 32.7% and 35.5% oligomannose-type glycans, respectively. It would be interesting to determine the glycosylation of Envs within the HIV-infected donor who transmitted the viruses to the CAP256 donor; however, these samples are not available.

Although we have studied only one HIV-infected individual in detail, a number of studies have shown that t/f viruses have a lower frequency of PNGSs (39–43). Whether there would be a benefit for t/f viruses to have a reduced frequency of PNGSs and subsequently to display a lower proportion of oligomannose-type glycans is unclear. In relation to HIV transmission, studies have shown that the importance of the interaction of DC-SIGN receptors on dendritic cells (DCs) in mucosal tissues for transfection of CD4^+^ T cells is strongly dependent on the presence of oligomannose structures (65–67). In relation to infectivity, reduction of complex-type glycans on HIV virions (through the use of glycosidase inhibitors or a GnTI-deficient cell line) reduced the infectivity of the virus but enhanced transinfection of peripheral blood lymphoctyes (32, 68). In relation to Env immunogenicity, studies have shown that removal or occlusion of mannose residues from the surface of gp120 can enhance the immune response against HIV due to reduced interactions with immunosuppressive receptors, such as the mannose receptor (69–71). Taken together, these studies might suggest that a higher abundance of oligomannose-type glycans would be more beneficial for transmitted viruses. It is therefore possible that the reduced oligomannose levels in the PI and SU viruses are only a consequence of fewer PNGSs and do not give a virus a competitive advantage at the point of transmission. However, there may be a tradeoff between viral infectivity and host recognition. Regardless, in terms of vaccine design, Env-based immunogens with a lower abundance of oligomannose-type glycans (for the CAP256 donor, this would be Envs from earlier time points) might give a stronger immune response, as suggested by the studies described above (69–71).

In summary, although in the CAP256 donor there were changes in both the frequency and positioning of PNGSs due to immune pressure, the intrinsic mannose patch remained a stable feature of HIV Env and was present throughout the course of HIV infection. The density of PNGSs on the outer domain of gp120 can influence the size and composition of the intrinsic mannose patch, but these differences do not affect the neutralization sensitivity of a panel of HIV bnAbs. These findings, in addition to our previous observations showing the presence of the intrinsic mannose patch to be independent of producer cells, further highlight the mannose patch as a stable target for HIV vaccine design.

## Supplementary Material

Supplemental material

## References

[1] Crispin M, Doores KJ 2015 Targeting host-derived glycans on enveloped viruses for antibody-based vaccine design. Curr Opin Virol 11:63–69. doi:10.1016/j.coviro.2015.02.002. 25747313 PMC4827424

[2] Doores KJ 2015 The HIV glycan shield as a target for broadly neutralizing antibodies. FEBS J 282:4679–4691. doi:10.1111/febs.13530. 26411545 PMC4950053

[3] Wei X, Decker JM, Wang S, Hui H, Kappes JC, Wu X, Salazar-Gonzalez JF, Salazar MG, Kilby JM, Saag MS, Komarova NL, Nowak MA, Hahn BH, Kwong PD, Shaw GM 2003 Antibody neutralization and escape by HIV-1. Nature 422:307–312. doi:10.1038/nature01470. 12646921

[4] Scanlan CN, Pantophlet R, Wormald MR, Ollmann Saphire E, Stanfield R, Wilson IA, Katinger H, Dwek RA, Rudd PM, Burton DR 2002 The broadly neutralizing anti-human immunodeficiency virus type 1 antibody 2G12 recognizes a cluster of alpha1→2 mannose residues on the outer face of gp120. J Virol 76:7306–7321. doi:10.1128/JVI.76.14.7306-7321.2002. 12072529 PMC136327

[5] Walker LM, Huber M, Doores KJ, Falkowska E, Pejchal R, Julien JP, Wang SK, Ramos A, Chan-Hui PY, Moyle M, Mitcham JL, Hammond PW, Olsen OA, Phung P, Fling S, Wong CH, Phogat S, Wrin T, Simek MD, Koff WC, Wilson IA, Burton DR, Poignard P 2011 Broad neutralization coverage of HIV by multiple highly potent antibodies. Nature 477:466–470. doi:10.1038/nature10373. 21849977 PMC3393110

[6] Scanlan CN, Offer J, Zitzmann N, Dwek RA 2007 Exploiting the defensive sugars of HIV-1 for drug and vaccine design. Nature 446:1038–1045. doi:10.1038/nature05818. 17460665

[7] Doores KJ, Bonomelli C, Harvey DJ, Vasiljevic S, Dwek RA, Burton DR, Crispin M, Scanlan CN 2010 Envelope glycans of immunodeficiency virions are almost entirely oligomannose antigens. Proc Natl Acad Sci U S A 107:13800–13805. doi:10.1073/pnas.1006498107. 20643940 PMC2922250

[8] Go EP, Irungu J, Zhang Y, Dalpathado DS, Liao HX, Sutherland LL, Alam SM, Haynes BF, Desaire H 2008 Glycosylation site-specific analysis of HIV envelope proteins (JR-FL and CON-S) reveals major differences in glycosylation site occupancy, glycoform profiles, and antigenic epitopes' accessibility. J Proteome Res 7:1660–1674. doi:10.1021/pr7006957. 18330979 PMC3658474

[9] Mizuochi T, Spellman MW, Larkin M, Solomon J, Basa LJ, Feizi T 1988 Structural characterization by chromatographic profiling of the oligosaccharides of human immunodeficiency virus (HIV) recombinant envelope glycoprotein gp120 produced in Chinese hamster ovary cells. Biomed Chromatogr 2:260–270. 2852980 10.1002/bmc.1130020608

[10] Mizuochi T, Spellman MW, Larkin M, Solomon J, Basa LJ, Feizi T 1988 Carbohydrate structures of the human-immunodeficiency-virus (HIV) recombinant envelope glycoprotein gp120 produced in Chinese-hamster ovary cells. Biochem J 254:599–603. doi:10.1042/bj2540599. 2845957 PMC1135120

[11] Zhu X, Borchers C, Bienstock RJ, Tomer KB 2000 Mass spectrometric characterization of the glycosylation pattern of HIV-gp120 expressed in CHO cells. Biochemistry 39:11194–11204. doi:10.1021/bi000432m. 10985765

[12] Mizuochi T, Matthews TJ, Kato M, Hamako J, Titani K, Solomon J, Feizi T 1990 Diversity of oligosaccharide structures on the envelope glycoprotein gp 120 of human immunodeficiency virus 1 from the lymphoblastoid cell line H9. Presence of complex-type oligosaccharides with bisecting N-acetylglucosamine residues. J Biol Chem 265:8519–8524. 2341393

[13] Geyer H, Holschbach C, Hunsmann G, Schneider J 1988 Carbohydrates of human immunodeficiency virus. Structures of oligosaccharides linked to the envelope glycoprotein 120. J Biol Chem 263:11760–11767. 2841333

[14] Leonard CK, Spellman MW, Riddle L, Harris RJ, Thomas JN, Gregory TJ 1990 Assignment of intrachain disulfide bonds and characterization of potential glycosylation sites of the type 1 recombinant human immunodeficiency virus envelope glycoprotein (gp120) expressed in Chinese hamster ovary cells. J Biol Chem 265:10373–10382. 2355006

[15] Bonomelli C, Doores KJ, Dunlop DC, Thaney V, Dwek RA, Burton DR, Crispin M, Scanlan CN 2011 The glycan shield of HIV is predominantly oligomannose independently of production system or viral clade. PLoS One 6:e23521. doi:10.1371/journal.pone.0023521. 21858152 PMC3156772

[16] Pritchard LK, Spencer DI, Royle L, Bonomelli C, Seabright GE, Behrens AJ, Kulp DW, Menis S, Krumm SA, Dunlop DC, Crispin DJ, Bowden TA, Scanlan CN, Ward AB, Schief WR, Doores KJ, Crispin M 2015 Glycan clustering stabilizes the mannose patch of HIV-1 and preserves vulnerability to broadly neutralizing antibodies. Nat Commun 6:7479. doi:10.1038/ncomms8479. 26105115 PMC4500839

[17] Pritchard LK, Harvey DJ, Bonomelli C, Crispin M, Doores KJ 2015 Cell- and protein-directed glycosylation of native cleaved HIV-1 envelope. J Virol 89:8932–8944. doi:10.1128/JVI.01190-15. 26085151 PMC4524065

[18] Pritchard LK, Vasiljevic S, Ozorowski G, Seabright GE, Cupo A, Ringe RP, Kim HJ, Sanders RW, Doores KJ, Burton DR, Wilson IA, Ward AB, Moore JP, Crispin M 2015 Structural constraints determine the glycosylation of HIV-1 envelope trimers. Cell Rep 11:1604–1613. doi:10.1016/j.celrep.2015.05.017. 26051934 PMC4555872

[19] Go EP, Liao HX, Alam SM, Hua D, Haynes BF, Desaire H 2013 Characterization of host-cell line specific glycosylation profiles of early transmitted/founder HIV-1 gp120 envelope proteins. J Proteome Res 12:1223–1234. doi:10.1021/pr300870t. 23339644 PMC3674872

[20] Go EP, Herschhorn A, Gu C, Castillo-Menendez L, Zhang S, Mao Y, Chen H, Ding H, Wakefield JK, Hua D, Liao HX, Kappes JC, Sodroski J, Desaire H 2015 Comparative analysis of the glycosylation profiles of membrane-anchored HIV-1 envelope glycoprotein trimers and soluble gp140. J Virol 89:8245–8257. doi:10.1128/JVI.00628-15. 26018173 PMC4524223

[21] Panico M, Bouche L, Binet D, O'Connor MJ, Rahman D, Pang PC, Canis K, North SJ, Desrosiers RC, Chertova E, Keele BF, Bess JW Jr, Lifson JD, Haslam SM, Dell A, Morris HR 2016 Mapping the complete glycoproteome of virion-derived HIV-1 gp120 provides insights into broadly neutralizing antibody binding. Sci Rep 6:32956. doi:10.1038/srep32956. 27604319 PMC5015092

[22] Behrens AJ, Vasiljevic S, Pritchard LK, Harvey DJ, Andev RS, Krumm SA, Struwe WB, Cupo A, Kumar A, Zitzmann N, Seabright GE, Kramer HB, Spencer DI, Royle L, Lee JH, Klasse PJ, Burton DR, Wilson IA, Ward AB, Sanders RW, Moore JP, Doores KJ, Crispin M 2016 Composition and antigenic effects of individual glycan sites of a trimeric HIV-1 envelope glycoprotein. Cell Rep 14:2695–2706. doi:10.1016/j.celrep.2016.02.058. 26972002 PMC4805854

[23] Kong L, Lee JH, Doores KJ, Murin CD, Julien JP, McBride R, Liu Y, Marozsan A, Cupo A, Klasse PJ, Hoffenberg S, Caulfield M, King CR, Hua Y, Le KM, Khayat R, Deller MC, Clayton T, Tien H, Feizi T, Sanders RW, Paulson JC, Moore JP, Stanfield RL, Burton DR, Ward AB, Wilson IA 2013 Supersite of immune vulnerability on the glycosylated face of HIV-1 envelope glycoprotein gp120. Nat Struct Mol Biol 20:796–803. doi:10.1038/nsmb.2594. 23708606 PMC3823233

[24] Mouquet H, Scharf L, Euler Z, Liu Y, Eden C, Scheid JF, Halper-Stromberg A, Gnanapragasam PN, Spencer DI, Seaman MS, Schuitemaker H, Feizi T, Nussenzweig MC, Bjorkman PJ 2012 Complex-type N-glycan recognition by potent broadly neutralizing HIV antibodies. Proc Natl Acad Sci U S A 109:E3268–E3277. doi:10.1073/pnas.1217207109. 23115339 PMC3511153

[25] Pejchal R, Doores KJ, Walker LM, Khayat R, Huang PS, Wang SK, Stanfield RL, Julien JP, Ramos A, Crispin M, Depetris R, Katpally U, Marozsan A, Cupo A, Maloveste S, Liu Y, McBride R, Ito Y, Sanders RW, Ogohara C, Paulson JC, Feizi T, Scanlan CN, Wong CH, Moore JP, Olson WC, Ward AB, Poignard P, Schief WR, Burton DR, Wilson IA 2011 A potent and broad neutralizing antibody recognizes and penetrates the HIV glycan shield. Science 334:1097–1103. doi:10.1126/science.1213256. 21998254 PMC3280215

[26] Walker LM, Phogat SK, Chan-Hui PY, Wagner D, Phung P, Goss JL, Wrin T, Simek MD, Fling S, Mitcham JL, Lehrman JK, Priddy FH, Olsen OA, Frey SM, Hammond PW, Kaminsky S, Zamb T, Moyle M, Koff WC, Poignard P, Burton DR 2009 Broad and potent neutralizing antibodies from an African donor reveal a new HIV-1 vaccine target. Science 326:285–289. doi:10.1126/science.1178746. 19729618 PMC3335270

[27] Doria-Rose NA, Bhiman JN, Roark RS, Schramm CA, Gorman J, Chuang GY, Pancera M, Cale EM, Ernandes MJ, Louder MK, Asokan M, Bailer RT, Druz A, Fraschilla IR, Garrett NJ, Jarosinski M, Lynch RM, McKee K, O'Dell S, Pegu A, Schmidt SD, Staupe RP, Sutton MS, Wang K, Wibmer CK, Haynes BF, Abdool-Karim S, Shapiro L, Kwong PD, Moore PL, Morris L, Mascola JR 2016 New member of the V1V2-directed CAP256-VRC26 lineage that shows increased breadth and exceptional potency. J Virol 90:76–91. doi:10.1128/JVI.01791-15. PMC4702551 26468542

[28] Bonsignori M, Hwang KK, Chen X, Tsao CY, Morris L, Gray E, Marshall DJ, Crump JA, Kapiga SH, Sam NE, Sinangil F, Pancera M, Yongping Y, Zhang B, Zhu J, Kwong PD, O'Dell S, Mascola JR, Wu L, Nabel GJ, Phogat S, Seaman MS, Whitesides JF, Moody MA, Kelsoe G, Yang X, Sodroski J, Shaw GM, Montefiori DC, Kepler TB, Tomaras GD, Alam SM, Liao HX, Haynes BF 2011 Analysis of a clonal lineage of HIV-1 envelope V2/V3 conformational epitope-specific broadly neutralizing antibodies and their inferred unmutated common ancestors. J Virol 85:9998–10009. doi:10.1128/JVI.05045-11. 21795340 PMC3196428

[29] Falkowska E, Le KM, Ramos A, Doores KJ, Lee JH, Blattner C, Ramirez A, Derking R, van Gils MJ, Liang CH, McBride R, von Bredow B, Shivatare SS, Wu CY, Chan-Hui PY, Liu Y, Feizi T, Zwick MB, Koff WC, Seaman MS, Swiderek K, Moore JP, Evans D, Paulson JC, Wong CH, Ward AB, Wilson IA, Sanders RW, Poignard P, Burton DR 2014 Broadly neutralizing HIV antibodies define a glycan-dependent epitope on the prefusion conformation of gp41 on cleaved envelope trimers. Immunity 40:657–668. doi:10.1016/j.immuni.2014.04.009. 24768347 PMC4070425

[30] Huang J, Kang BH, Pancera M, Lee JH, Tong T, Feng Y, Imamichi H, Georgiev IS, Chuang GY, Druz A, Doria-Rose NA, Laub L, Sliepen K, van Gils MJ, de la Pena AT, Derking R, Klasse PJ, Migueles SA, Bailer RT, Alam M, Pugach P, Haynes BF, Wyatt RT, Sanders RW, Binley JM, Ward AB, Mascola JR, Kwong PD, Connors M 2014 Broad and potent HIV-1 neutralization by a human antibody that binds the gp41-gp120 interface. Nature 515:138–142. doi:10.1038/nature13601. 25186731 PMC4224615

[31] Scharf L, Scheid JF, Lee JH, West AP Jr, Chen C, Gao H, Gnanapragasam PN, Mares R, Seaman MS, Ward AB, Nussenzweig MC, Bjorkman PJ 2014 Antibody 8ANC195 reveals a site of broad vulnerability on the HIV-1 envelope spike. Cell Rep 7:785–795. doi:10.1016/j.celrep.2014.04.001. 24767986 PMC4109818

[32] Binley JM, Ban YE, Crooks ET, Eggink D, Osawa K, Schief WR, Sanders RW 2010 Role of complex carbohydrates in human immunodeficiency virus type 1 infection and resistance to antibody neutralization. J Virol 84:5637–5655. doi:10.1128/JVI.00105-10. 20335257 PMC2876609

[33] Kim AS, Leaman DP, Zwick MB 2014 Antibody to gp41 MPER alters functional properties of HIV-1 Env without complete neutralization. PLoS Pathog 10:e1004271. doi:10.1371/journal.ppat.1004271. 25058619 PMC4110039

[34] Reitter JN, Means RE, Desrosiers RC 1998 A role for carbohydrates in immune evasion in AIDS. Nat Med 4:679–684. doi:10.1038/nm0698-679. 9623976

[35] Moore PL, Gray ES, Wibmer CK, Bhiman JN, Nonyane M, Sheward DJ, Hermanus T, Bajimaya S, Tumba NL, Abrahams MR, Lambson BE, Ranchobe N, Ping L, Ngandu N, Abdool Karim Q, Abdool Karim SS, Swanstrom RI, Seaman MS, Williamson C, Morris L 2012 Evolution of an HIV glycan-dependent broadly neutralizing antibody epitope through immune escape. Nat Med 18:1688–1692. doi:10.1038/nm.2985. 23086475 PMC3494733

[36] Gao F, Bonsignori M, Liao HX, Kumar A, Xia SM, Lu X, Cai F, Hwang KK, Song H, Zhou T, Lynch RM, Alam SM, Moody MA, Ferrari G, Berrong M, Kelsoe G, Shaw GM, Hahn BH, Montefiori DC, Kamanga G, Cohen MS, Hraber P, Kwong PD, Korber BT, Mascola JR, Kepler TB, Haynes BF 2014 Cooperation of B cell lineages in induction of HIV-1-broadly neutralizing antibodies. Cell 158:481–491. doi:10.1016/j.cell.2014.06.022. 25065977 PMC4150607

[37] Wibmer CK, Bhiman JN, Gray ES, Tumba N, Abdool Karim SS, Williamson C, Morris L, Moore PL 2013 Viral escape from HIV-1 neutralizing antibodies drives increased plasma neutralization breadth through sequential recognition of multiple epitopes and immunotypes. PLoS Pathog 9:e1003738. doi:10.1371/journal.ppat.1003738. 24204277 PMC3814426

[38] McGuire AT, Hoot S, Dreyer AM, Lippy A, Stuart A, Cohen KW, Jardine J, Menis S, Scheid JF, West AP, Schief WR, Stamatatos L 2013 Engineering HIV envelope protein to activate germline B cell receptors of broadly neutralizing anti-CD4 binding site antibodies. J Exp Med 210:655–663. doi:10.1084/jem.20122824. 23530120 PMC3620356

[39] Derdeyn CA, Decker JM, Bibollet-Ruche F, Mokili JL, Muldoon M, Denham SA, Heil ML, Kasolo F, Musonda R, Hahn BH, Shaw GM, Korber BT, Allen S, Hunter E 2004 Envelope-constrained neutralization-sensitive HIV-1 after heterosexual transmission. Science 303:2019–2022. doi:10.1126/science.1093137. 15044802

[40] Chohan B, Lang D, Sagar M, Korber B, Lavreys L, Richardson B, Overbaugh J 2005 Selection for human immunodeficiency virus type 1 envelope glycosylation variants with shorter V1-V2 loop sequences occurs during transmission of certain genetic subtypes and may impact viral RNA levels. J Virol 79:6528–6531. doi:10.1128/JVI.79.10.6528-6531.2005. 15858037 PMC1091724

[41] Edo-Matas D, Rachinger A, Setiawan LC, Boeser-Nunnink BD, van 't Wout AB, Lemey P, Schuitemaker H 2012 The evolution of human immunodeficiency virus type-1 (HIV-1) envelope molecular properties and coreceptor use at all stages of infection in an HIV-1 donor-recipient pair. Virology 422:70–80. doi:10.1016/j.virol.2011.10.005. 22047989

[42] Parrish NF, Gao F, Li H, Giorgi EE, Barbian HJ, Parrish EH, Zajic L, Iyer SS, Decker JM, Kumar A, Hora B, Berg A, Cai F, Hopper J, Denny TN, Ding H, Ochsenbauer C, Kappes JC, Galimidi RP, West AP Jr, Bjorkman PJ, Wilen CB, Doms RW, O'Brien M, Bhardwaj N, Borrow P, Haynes BF, Muldoon M, Theiler JP, Korber B, Shaw GM, Hahn BH 2013 Phenotypic properties of transmitted founder HIV-1. Proc Natl Acad Sci U S A 110:6626–6633. doi:10.1073/pnas.1304288110. 23542380 PMC3637789

[43] Samleerat T, Braibant M, Jourdain G, Moreau A, Ngo-Giang-Huong N, Leechanachai P, Hemvuttiphan J, Hinjiranandana T, Changchit T, Warachit B, Suraseranivong V, Lallemant M, Barin F 2008 Characteristics of HIV type 1 (HIV-1) glycoprotein 120 env sequences in mother-infant pairs infected with HIV-1 subtype CRF01_AE. J Infect Dis 198:868–876. doi:10.1086/591251. 18700833

[44] Curlin ME, Zioni R, Hawes SE, Liu Y, Deng W, Gottlieb GS, Zhu T, Mullins JI 2010 HIV-1 envelope subregion length variation during disease progression. PLoS Pathog 6:e1001228. doi:10.1371/journal.ppat.1001228. 21187897 PMC3002983

[45] Bunnik EM, Pisas L, van Nuenen AC, Schuitemaker H 2008 Autologous neutralizing humoral immunity and evolution of the viral envelope in the course of subtype B human immunodeficiency virus type 1 infection. J Virol 82:7932–7941. doi:10.1128/JVI.00757-08. 18524815 PMC2519599

[46] Chackerian B, Rudensey LM, Overbaugh J 1997 Specific N-linked and O-linked glycosylation modifications in the envelope V1 domain of simian immunodeficiency virus variants that evolve in the host alter recognition by neutralizing antibodies. J Virol 71:7719–7727. 9311856 10.1128/jvi.71.10.7719-7727.1997 PMC192123

[47] Back NK, Smit L, De Jong JJ, Keulen W, Schutten M, Goudsmit J, Tersmette M 1994 An N-glycan within the human immunodeficiency virus type 1 gp120 V3 loop affects virus neutralization. Virology 199:431–438. doi:10.1006/viro.1994.1141. 8122371

[48] Doria-Rose NA, Schramm CA, Gorman J, Moore PL, Bhiman JN, DeKosky BJ, Ernandes MJ, Georgiev IS, Kim HJ, Pancera M, Staupe RP, Altae-Tran HR, Bailer RT, Crooks ET, Cupo A, Druz A, Garrett NJ, Hoi KH, Kong R, Louder MK, Longo NS, McKee K, Nonyane M, O'Dell S, Roark RS, Rudicell RS, Schmidt SD, Sheward DJ, Soto C, Wibmer CK, Yang Y, Zhang Z, Mullikin JC, Binley JM, Sanders RW, Wilson IA, Moore JP, Ward AB, Georgiou G, Williamson C, Abdool Karim SS, Morris L, Kwong PD, Shapiro L, Mascola JR 2014 Developmental pathway for potent V1V2-directed HIV-neutralizing antibodies. Nature 509:55–62. doi:10.1038/nature13036. 24590074 PMC4395007

[49] Moore PL, Sheward D, Nonyane M, Ranchobe N, Hermanus T, Gray ES, Abdool Karim SS, Williamson C, Morris L 2013 Multiple pathways of escape from HIV broadly cross-neutralizing V2-dependent antibodies. J Virol 87:4882–4894. doi:10.1128/JVI.03424-12. 23408621 PMC3624332

[50] Aricescu AR, Lu W, Jones EY 2006 A time- and cost-efficient system for high-level protein production in mammalian cells. Acta Crystallogr D Biol Crystallogr 62:1243–1250. doi:10.1107/S0907444906029799. 17001101

[51] Dunlop DC, Bonomelli C, Mansab F, Vasiljevic S, Doores KJ, Wormald MR, Palma AS, Feizi T, Harvey DJ, Dwek RA, Crispin M, Scanlan CN 2010 Polysaccharide mimicry of the epitope of the broadly neutralizing anti-HIV antibody, 2G12, induces enhanced antibody responses to self oligomannose glycans. Glycobiology 20:812–823. doi:10.1093/glycob/cwq020. 20181792 PMC2900896

[52] Royle L, Radcliffe CM, Dwek RA, Rudd PM 2006 Detailed structural analysis of N-glycans released from glycoproteins in SDS-PAGE gel bands using HPLC combined with exoglycosidase array digestions. Methods Mol Biol 347:125–143. 17072008 10.1385/1-59745-167-3:125

[53] Neville DC, Dwek RA, Butters TD 2009 Development of a single column method for the separation of lipid- and protein-derived oligosaccharides. J Proteome Res 8:681–687. doi:10.1021/pr800704t. 19099509

[54] Li M, Gao F, Mascola JR, Stamatatos L, Polonis VR, Koutsoukos M, Voss G, Goepfert P, Gilbert P, Greene KM, Bilska M, Kothe DL, Salazar-Gonzalez JF, Wei X, Decker JM, Hahn BH, Montefiori DC 2005 Human immunodeficiency virus type 1 env clones from acute and early subtype B infections for standardized assessments of vaccine-elicited neutralizing antibodies. J Virol 79:10108–10125. doi:10.1128/JVI.79.16.10108-10125.2005. 16051804 PMC1182643

[55] Montefiori DC 2005 Evaluating neutralizing antibodies against HIV, SIV, and SHIV in luciferase reporter gene assays. Curr Protoc Immunol Chapter 12:Unit 12.11. 10.1002/0471142735.im1211s64 18432938

[56] Moore PL, Gray ES, Choge IA, Ranchobe N, Mlisana K, Abdool Karim SS, Williamson C, Morris L, Team CS 2008 The c3-v4 region is a major target of autologous neutralizing antibodies in human immunodeficiency virus type 1 subtype C infection. J Virol 82:1860–1869. doi:10.1128/JVI.02187-07. 18057243 PMC2258729

[57] Bhiman JN, Anthony C, Doria-Rose NA, Karimanzira O, Schramm CA, Khoza T, Kitchin D, Botha G, Gorman J, Garrett NJ, Abdool Karim SS, Shapiro L, Williamson C, Kwong PD, Mascola JR, Morris L, Moore PL 2015 Viral variants that initiate and drive maturation of V1V2-directed HIV-1 broadly neutralizing antibodies. Nat Med 21:1332–1336. doi:10.1038/nm.3963. 26457756 PMC4637988

[58] Julien JP, Cupo A, Sok D, Stanfield RL, Lyumkis D, Deller MC, Klasse PJ, Burton DR, Sanders RW, Moore JP, Ward AB, Wilson IA 2013 Crystal structure of a soluble cleaved HIV-1 envelope trimer. Science 342:1477–1483. doi:10.1126/science.1245625. 24179159 PMC3886632

[59] Julien JP, Lee JH, Cupo A, Murin CD, Derking R, Hoffenberg S, Caulfield MJ, King CR, Marozsan AJ, Klasse PJ, Sanders RW, Moore JP, Wilson IA, Ward AB 2013 Asymmetric recognition of the HIV-1 trimer by broadly neutralizing antibody PG9. Proc Natl Acad Sci U S A 110:4351–4356. doi:10.1073/pnas.1217537110. 23426631 PMC3600498

[60] Go EP, Hewawasam G, Liao HX, Chen H, Ping LH, Anderson JA, Hua DC, Haynes BF, Desaire H 2011 Characterization of glycosylation profiles of HIV-1 transmitted/founder envelopes by mass spectrometry. J Virol 85:8270–8284. doi:10.1128/JVI.05053-11. 21653661 PMC3147976

[61] Pancera M, Zhou T, Druz A, Georgiev IS, Soto C, Gorman J, Huang J, Acharya P, Chuang GY, Ofek G, Stewart-Jones GB, Stuckey J, Bailer RT, Joyce MG, Louder MK, Tumba N, Yang Y, Zhang B, Cohen MS, Haynes BF, Mascola JR, Morris L, Munro JB, Blanchard SC, Mothes W, Connors M, Kwong PD 2014 Structure and immune recognition of trimeric pre-fusion HIV-1 Env. Nature 514:455–461. doi:10.1038/nature13808. 25296255 PMC4348022

[62] Gray ES, Moore PL, Pantophlet RA, Morris L 2007 N-linked glycan modifications in gp120 of human immunodeficiency virus type 1 subtype C render partial sensitivity to 2G12 antibody neutralization. J Virol 81:10769–10776. doi:10.1128/JVI.01106-07. 17634239 PMC2045459

[63] Sanders RW, Venturi M, Schiffner L, Kalyanaraman R, Katinger H, Lloyd KO, Kwong PD, Moore JP 2002 The mannose-dependent epitope for neutralizing antibody 2G12 on human immunodeficiency virus type 1 glycoprotein gp120. J Virol 76:7293–7305. doi:10.1128/JVI.76.14.7293-7305.2002. 12072528 PMC136300

[64] Pritchard LK, Spencer DI, Royle L, Vasiljevic S, Krumm SA, Doores KJ, Crispin M 2015 Glycan microheterogeneity at the PGT135 antibody recognition site on HIV-1 gp120 reveals a molecular mechanism for neutralization resistance. J Virol 89:6952–6959. doi:10.1128/JVI.00230-15. 25878100 PMC4468474

[65] Geijtenbeek TB, Kwon DS, Torensma R, van Vliet SJ, van Duijnhoven GC, Middel J, Cornelissen IL, Nottet HS, KewalRamani VN, Littman DR, Figdor CG, van Kooyk Y 2000 DC-SIGN, a dendritic cell-specific HIV-1-binding protein that enhances trans-infection of T cells. Cell 100:587–597. doi:10.1016/S0092-8674(00)80694-7. 10721995

[66] Hong PW, Flummerfelt KB, de Parseval A, Gurney K, Elder JH, Lee B 2002 Human immunodeficiency virus envelope (gp120) binding to DC-SIGN and primary dendritic cells is carbohydrate dependent but does not involve 2G12 or cyanovirin binding sites: implications for structural analyses of gp120-DC-SIGN binding. J Virol 76:12855–12865. doi:10.1128/JVI.76.24.12855-12865.2002. 12438611 PMC136699

[67] Kwon DS, Gregorio G, Bitton N, Hendrickson WA, Littman DR 2002 DC-SIGN-mediated internalization of HIV is required for trans-enhancement of T cell infection. Immunity 16:135–144. doi:10.1016/S1074-7613(02)00259-5. 11825572

[68] Shen R, Raska M, Bimczok D, Novak J, Smith PD 2014 HIV-1 envelope glycan moieties modulate HIV-1 transmission. J Virol 88:14258–14267. doi:10.1128/JVI.02164-14. 25275130 PMC4249159

[69] Banerjee K, Andjelic S, Klasse PJ, Kang Y, Sanders RW, Michael E, Durso RJ, Ketas TJ, Olson WC, Moore JP 2009 Enzymatic removal of mannose moieties can increase the immune response to HIV-1 gp120 in vivo. Virology 389:108–121. doi:10.1016/j.virol.2009.04.001. 19410272 PMC2743082

[70] Banerjee K, Michael E, Eggink D, van Montfort T, Lasnik AB, Palmer KE, Sanders RW, Moore JP, Klasse PJ 2012 Occluding the mannose moieties on human immunodeficiency virus type 1 gp120 with griffithsin improves the antibody responses to both proteins in mice. AIDS Res Hum Retroviruses 28:206–214. doi:10.1089/aid.2011.0101. 21793733 PMC3275927

[71] Kong L, Sheppard NC, Stewart-Jones GB, Robson CL, Chen H, Xu X, Krashias G, Bonomelli C, Scanlan CN, Kwong PD, Jeffs SA, Jones IM, Sattentau QJ 2010 Expression-system-dependent modulation of HIV-1 envelope glycoprotein antigenicity and immunogenicity. J Mol Biol 403:131–147. doi:10.1016/j.jmb.2010.08.033. 20800070 PMC2950005

[72] Lee JH, de Val N, Lyumkis D, Ward AB 2015 Model building and refinement of a natively glycosylated HIV-1 Env protein by high-resolution cryoelectron microscopy. Structure 23:1943–1951. doi:10.1016/j.str.2015.07.020. 26388028 PMC4618500

