# Supplementary material for: HIV-1 Glycan Density Drives the Persistence of the Mannose Patch within an Infected Individual
Source: J Virol. 2016 Nov 28;90(24):11132–44. doi: 10.1128/JVI.01542-16 (PMC5126371; doi:10.1128/JVI.01542-16)
Supplement: Supplemental material [file supp_90_24_11132__index.html]

Supplemental material 

# HIV-1 Glycan Density Drives the Persistence of the Mannose Patch within an Infected Individual

## Supplemental material

**Files in this Data Supplement:**

- Supplemental file 1 -

  Table S1 (Abundance of oligomannose-type glycans for each CAP256 gp120.) Table S2 (Abundance of oligomannose-type glycans for a cross-clade panel of gp120s.) Table S3 (Potency of neutralizaAon for a panel of HIV bnAb against the CAP256 pseudoviruses.)

  PDF, 153K
