# Supplementary material for: HIV-1 Glycan Density Drives the Persistence of the Mannose Patch within an Infected Individual
Source: J Virol. 2016 Nov 28;90(24):11132–44. doi: 10.1128/JVI.01542-16 (PMC5126371; doi:10.1128/JVI.01542-16)
Supplement: Supplemental material [file JVI.01542-16_zjv999182187s1.pdf]

Table S1: Abundance of oligomannose-type glycans for each CAP256 gp120. A breakdown of the distribution of structures within the oligomannose series ($Man_{5-9}GlcNAc_2$) are also reported. We have previously shown a coefficient of variance of 1.5 (mean 36.7, SD 0.6) for the assessment of the oligomannose series on $gp120_{BaL}$ expressed and analysed 5 times in parallel (Pritchard et al, Nat. Commun, 2015, 6:7479). Therefore a 4% variation in Man_5-$Man_9GlcNAc_2$ would represent ~6.6 SDs away from the mean and is therefore noteworthy.
Percentage change in $Man_{5-9}GlcNAc_2$ and $Man_9GlcNAc_2$ compared to the SU virus for each CAP256 gp120 clone studied are calculated using the formula: % change = ((%CAP-%SU)/%SU)*100).

Virus sequence	Time after infection (Weeks)	OD PNGSs	gp160 PNGSs	gp120 PNGSs	M5	M6	M7	M8	M9	Total (M5-M9)	% change in M9 compared to 15wks_SU	% change in M5-M9 compared to 15wks_SU
6wks_PI	6	13	27	23	10.6	4.3	6.8	8.7	4.9	35.3	-46.2	-3.0
15wks_SU	15	14	29	25	6.9	3.6	6.9	9.9	9.1	36.4	0.0	0.0
23wks.14	23	14	29	25	9.6	4.3	9.1	11.2	5.6	39.7	-38.5	9.1
23wks.13	23	15	30	26	8.7	3.7	8.3	12.9	12.4	46.0	36.3	26.4
30wks.2	30	14	29	25	6.5	3.0	5.4	10.4	8.4	33.7	-7.7	-7.3
34wks.22	34	14	27	23	9.1	5.0	10.1	13.4	5.3	42.9	-41.8	17.9
34wks.31	34	14	28	24	4.9	3.6	4.9	9.0	6.9	29.3	-24.1	-19.6
38wks.16	38	14	28	24	9.1	6.5	9.4	11.4	3.1	39.5	-65.9	8.5
38wks.19	38	14	29	25	8.1	4.0	7.9	12.5	5.7	38.1	-37.4	4.7
38wks.38	38	14	29	25	3.6	4.1	7.7	11.6	5.6	32.7	-38.5	-10.2
48wks.8	48	14	29	25	8.1	4.1	8.2	13.4	7.9	41.7	-13.2	14.6
48wks.10	48	14	28	24	8.2	3.1	7.0	8.3	8.8	35.5	-3.3	-2.5
48wks.17	48	15	28	24	8.7	4.1	7.7	12.7	10.7	43.8	17.6	20.3
48wks.18	48	15	30	26	4.9	6.2	9.5	14.4	7.0	42.0	-23.1	15.4
59wks.2a	59	15	30	26	8.9	4.0	7.7	9.9	9.9	40.4	8.8	11.0
59wks.4a	59	15	31	27	6.2	4.6	6.3	12.2	18.2	47.6	100.0	30.7
59wks.10b	59	15	30	26	5.8	3.9	6.0	12.0	16.4	44.0	79.9	20.9
94wks.A3	94	15	32	28	7.4	3.1	5.9	13.2	15.3	44.9	68.1	23.4
94wks.F4	94	15	30	26	5.3	4.2	6.0	13.4	15.7	44.7	73.0	22.7
176wks.C2	176	14	28	24	6.3	13.3	10.5	6.6	4.3	41.2	-52.7	13.2
176wks.H1	176	13	30	26	4.6	2.6	6.7	14.1	7.3	35.1	-19.8	-3.6
176wks.F1	176	13	28	24	5.6	3.1	6.4	11.7	6.5	33.3	-28.6	-8.5
176wks.4	176	14	31	27	1.8	7.8	10.9	10.4	7.1	38.2	-22.0	4.9
176wks.10	176	15	31	27	5.9	4.5	6.0	11.6	17.3	45.3	90.1	24.4

Table S2: Abundance of oligomannose-type glycans for a cross-clade panel of gp120s. A breakdown of the distribution of structures within the oligomannose series ($Man_{5-9}GlcNAc_2$) are also reported in addition to the total PNGSs on gp120 and on the outer domain. Gp120s are grouped together according to their clade. Transmitter/founder viruses are labeled T/F.

	Clade	M5	M6	M7	M8	M9	Total	Total PNGSs	OD PNGSs
**0260.V5.C36**	A	6.7	5.8	5.7	9.5	14.3	42.0	27	16
**191084 B7.19**	A (T/F)	5.5	6.8	9.9	11.7	7.9	41.7	25	14
**Q23-17**	A	9.1	7.2	7.1	9.4	5.4	38.3	23	15
**BG505**	A	7.4	5.5	8.7	11.8	9.7	43.1	24	15
**94UG103**	A	8.0	7.4	8.2	8.9	14.2	46.7	27	16
**92RW020**	A	4.1	4.6	7.5	11.2	7.7	35.1	25	16
**Q842**	A	5.6	5.7	6.7	6.2	2.3	26.6	23	15
**BaL**	B	2.5	3.1	6.2	10.7	11.6	34.1	23	14
**JRCSF**	B	2.8	3.7	6.6	7.8	8.5	29.4	23	14
**HXB2**	B	5.5	4.7	8.2	12.0	10.3	40.7	24	14
**SF162**	B	5.7	4.2	7.9	13.6	12.8	44.2	21	14
**TRJO_A2**	B	8.2	6.2	7.9	11.9	6.6	40.8	27	15
**1056_TA11_1826**	B (T/F)	5.4	5.7	7.4	9.1	8.0	35.4	25	15
**1012_11_TC21**	B (T/F)	5.5	5.5	8.5	11.8	9.9	41.2	28	15
**JRFL**	B	5.4	6.3	6.9	11.3	12.1	42.0	23	14
**96ZM651**	C	4.8	5.5	7.6	6.6	4.5	29.0	27	12
**Du156**	C	6.7	6.0	7.1	9.1	8.0	37.0	24	14
**ZM247v1(Rev-)**	C	9.2	6.9	6.7	11.7	14.9	49.3	27	16
**ZM214M_SGA_A3**	C	4.3	4.2	5.4	7.2	2.7	23.8	23	12
**IAVI C22**	C	5.8	5.7	10.3	11.4	5.9	39.2	24	15
**Ce7084**	C (T/F)	9.4	7.9	7.7	6.8	2.9	34.7	27	14
**C1080.B2**	AE	6.1	5.5	7.7	14.5	12.4	46.2	23	14
**92TH021**	AE	7.3	6.3	8.5	11.6	8.5	42.1	24	14
**BJOX015000.11.5**	AE (T/F)	7.8	6.2	7.3	10.3	5.8	37.3	25	14
**C3347**	AE	5.4	4.8	6.6	10.0	6.1	32.8	24	13
**P0402_c2_11**	G	10.6	8.8	9.3	11.6	6.0	46.3	27	16
**X1193_c1**	G	7.2	7.4	9.3	15.0	11.7	50.5	27	15
**X1254_3**	G	5.3	5.1	6.9	10.1	5.7	33.0	25	14
**X2088**	G	7.0	7.3	7.4	7.7	4.4	33.7	27	17

	M5	M6	M7	M8	M9	TOTAL	Total PNGS	OD PNGSs
**A**	6.6	6.1	7.7	9.8	8.8	39.1	24.9	15.3
**B**	5.1	4.9	7.5	11.0	10.0	38.5	24.3	14.4
**C**	6.7	6.0	7.5	8.8	6.5	35.5	25.3	13.8
**AE**	6.6	5.7	7.5	11.6	8.2	39.6	24.0	13.8
**G**	7.5	7.2	8.2	11.1	7.0	40.9	26.5	15.5

Table S3: Potency of neutralization for a panel of HIV bnAb against the CAP256 pseudoviruses. Values are reported as IC_{50} in μg/mL and colour coded according to the key.

Virus sequence	Time after infection (Weeks)	VRC01	PGV04	J3	PGT121	PGT128	PGT135	PGT151	PG9	CAP256-VRC26.06	CAP256-VRC26.27	CAP256-VRC26.25	CAP256-VRC26.11
6wks_PI	6	0.050	0.056	0.307	0.047	0.028	0.202	0.004	>10	0.03	>50	1	>50
15wks_SU	15	0.338	0.529	0.082	0.010	0.009	>20	>5	0.012	0.063	0.001	0.001	0.014
23wks.14	23	0.410	0.500	0.028	0.004	0.035	>20	>5	>10	0.11	>50	>50	>50
23wks.13	23	0.246	1.671	0.037	0.019	0.066	>20	>5	>10	0.086	>50	>50	>50
30wks.2	30	0.648	0.453	0.040	0.019	0.015	>20	>5	>10	0.006	>50	>50	11.3
34wks.22	34	0.600	0.790	0.027	0.005	0.021	>20	>5	>10	0.02	1.58	10	>50
34wks.31	34	0.529	1.268	0.065	0.004	0.002	>20	>5	0.051	0.2	0.010	0.001	0.04
38wks.16	38	0.815	0.770	0.046	0.017	0.052	>20	>5	>10	0.6	>50	>50	24
38wks.19	38	0.192	0.974	0.061	0.026	0.049	>20	0.021	>10	0.18	>50	>50	>50
38wks.38	38	0.265	0.945	0.080	0.045	>1	>20	0.009	>10	0.19	20.9	5	>50
48wks.8	48	0.634	0.910	0.190	0.005	0.046	>20	0.005	0.102	0.24	0.020	0.02	0.49
48wks.10	48	0.420	0.320	0.100	0.003	0.006	>20	0.005	0.378	>50	0.020	0.02	0.27
48wks.17	48	0.669	2.447	0.081	0.016	0.009	>20	>5	>10	>50	>50	>50	>50
48wks.18	48	4.483	1.711	0.242	0.015	0.005	>20	0.015	0.184	1.1	3.98	0.02	0.24
59wks.2a	59	0.549	0.468	0.079	0.044	0.011	>20	0.015	>10	8.9	>50	>50	1.7
59wks.4a	59	0.332	0.684	0.089	0.027	0.029	>20	>5	0.660	>50	>50	>50	>50
59wks.10b	59	0.764	0.501	0.033	0.009	0.011	>20	0.020	0.169	0.2	0.020	0.02	0.54
94wks.A3	94	0.311	0.519	0.041	0.033	0.016	0.029	0.005	1.49	>50	>50	>50	>50
94wks.F4	94	0.521	0.688	0.044	0.026	0.047	0.009	0.008	2.16	>50	>50	>50	>50
176wks.C2	176	0.106	0.088	0.061	0.055	>1	>20	0.002	>10	>50	>50	>50	>50
176wks.H1	176	0.161	0.255	0.105	>5	>1	>20	0.004	1.45	>50	>50	>50	>50
176wks.F1	176	0.065	0.170	0.024	0.217	>1	>20	0.002	>10	>50	>50	>50	>50
176wks.4	176	0.599	3.083	0.080	>5	>1	>20	0.009	>10	>50	>50	>50	>50
176wks.10	176	0.546	0.425	0.061	0.204	0.010	>20	0.008	>10	>50	>50	>50	>50

IC_{50} (ug/mL)
<0.01
0.01-0.1
>0.1-1
>1
